# The Protective Roles of Estrogen Receptor *β* in Renal Calcium Oxalate Crystal Formation *via* Reducing the Liver Oxalate Biosynthesis and Renal Oxidative Stress-Mediated Cell Injury

**DOI:** 10.1155/2019/5305014

**Published:** 2019-04-17

**Authors:** Wei Zhu, Zhijian Zhao, Fu-Ju Chou, Li Zuo, Tongzu Liu, David Bushinsky, Chawnshang Chang, Guohua Zeng, Shuyuan Yeh

**Affiliations:** ^1^Department of Urology and Guangdong Key Lab of Urology, The First Affiliated Hospital of Guangzhou Medical University, Guangzhou 510230, China; ^2^Departments of Urology and Pathology, University of Rochester Medical Center, Rochester, NY 14642, USA; ^3^Department of Medicine, University of Rochester Medical Center, Rochester, NY 14642, USA

## Abstract

Females develop kidney stones less frequently than males do. However, it is unclear if this gender difference is related to altered estrogen/estrogen receptor (ER) signaling. Here, we found that ER beta (ER*β*) signals could suppress hepatic oxalate biosynthesis *via* transcriptional upregulation of the glyoxylate aminotransferase (AGT1) expression. Results from multiple *in vitro* renal cell lines also found that ER*β* could function *via* suppressing the oxalate-induced injury through increasing the reactive oxygen species (ROS) production that led to a decrease of the renal calcium oxalate (CaOx) crystal deposition. Mechanism study results showed that ER*β* suppressed oxalate-induced oxidative stress *via* transcriptional suppression of the NADPH oxidase subunit 2 (NOX2) through direct binding to the estrogen response elements (EREs) on the NOX2 5′ promoter. We further applied two *in vivo* mouse models with glyoxylate-induced renal CaOx crystal deposition and one rat model with 5% hydroxyl-L-proline-induced renal CaOx crystal deposition. Our data demonstrated that mice lacking ER*β* (ER*β*KO) as well as mice or rats treated with ER*β* antagonist PHTPP had increased renal CaOx crystal deposition with increased urinary oxalate excretion and renal ROS production. Importantly, targeting ER*β*-regulated NOX2 with the NADPH oxidase inhibitor, apocynin, can suppress the renal CaOx crystal deposition in the *in vivo* mouse model. Together, results from multiple *in vitro* cell lines and *in vivo* mouse/rat models all demonstrate that ER*β* may protect against renal CaOx crystal deposition *via* inhibiting the hepatic oxalate biosynthesis and oxidative stress-induced renal injury.

## 1. Introduction

Kidney stone is a common disorder that poses a significant health care burden in a working-age population [1]. The incidence of kidney stone disease is lower in premenopausal women than age-matched men, as well as in female animal models with renal calcium oxalate (CaOx) crystal deposition [2–6]. However, this disparity becomes less prominent in the sixth decade of life in parallel with the onset of menopause in women [7, 8]. These studies may indicate a protective function of 17*β*-estradiol (E2) in the kidney stone formation. However, results from the Women's Health Initiative (NHI) study and Nurse's Health Study all failed to find a positive linkage of hormone replacement therapy (HRT) with the kidney stone prevention [7–9]. It is still not clear why the beneficial effects of HRT were lost in these postmenopausal females. Turner and Kerber [9] proposed that long periods of hypoestrogenicity due to menopause may exacerbate the deterioration of the normal physiological ER function in the kidney, which may make “re-estrogenization”, by HRT, less beneficial. The possible reasons could be the reduced protein expressions of estrogen receptors (ERs) or their accessory factors in these women.

Estrogen actions are mediated mainly by two classical ER subtypes, ER*α* and ER*β*. Both ER*α* and ER*β* belong to the nuclear receptor superfamily and are genetically and functionally distinct [10]. Distribution of ER*α* and ER*β* also varies in different tissues and may play distinct roles in various disorders including hypertension [11] and brain injury [12]. At present, there is limited knowledge whether E2 may alter the development of kidney stones, and if it does, it remains unclear whether ER*α*, ER*β*, or both may be involved. In addition to inducing the nuclear genomic function, estrogens may induce rapid nongenomic responses from membrane-associated receptors, such as growth factor receptors and G protein-coupled estrogen receptor [13]. It remains to be investigated how E2 and its receptors affect the kidney stone formation.

The results from our previous nationwide cross-sectional survey on the prevalence of kidney stone formers in China of 3792 men and 5518 women revealed that the prevalence of kidney stone formers was 6.6% for men and 5.2% for women, suggesting that the gender difference existed among the kidney stone formers [14], with a lower incidence of kidney stone formers in the females. Together, all animal study data and clinical data analyses suggest that estrogens and ERs may play critical roles in renal CaOx crystal formation; however, the underlying mechanisms remain largely unclear.

CaOx kidney stones are the most prevalent solid-phase stones with a recurrence rate of approximately 40% at 5 years after the first treatment [15]. Hyperoxaluria plays key roles in CaOx kidney stone formation. The liver is the main organ of endogenous oxalate synthesis, in which glyoxylate aminotransferase (AGT1), glyoxylate aminotransferase 2 (AGT2), and glycolate oxidase (GO) are involved [16]. In addition, an increase of reactive oxygen species (ROS) and NADPH oxidase in the kidney gives rise to inflammation and injury of renal tubular cells, which may promote CaOx crystal formation [17].

Using multiple *in vitro* cell studies and *in vivo* mouse/rat models, here we found that ER*β* could play a protective role in suppressing renal CaOx crystal deposition *via* inhibiting the hepatic oxalate biosynthesis and oxidative stress-induced renal cell injury.

## 2. Results

### 2.1. ER*β* Decreases Hepatic Oxalate Biosynthesis *via* Increasing AGT1 Expression in Hepatocytes

**Figure 1:**
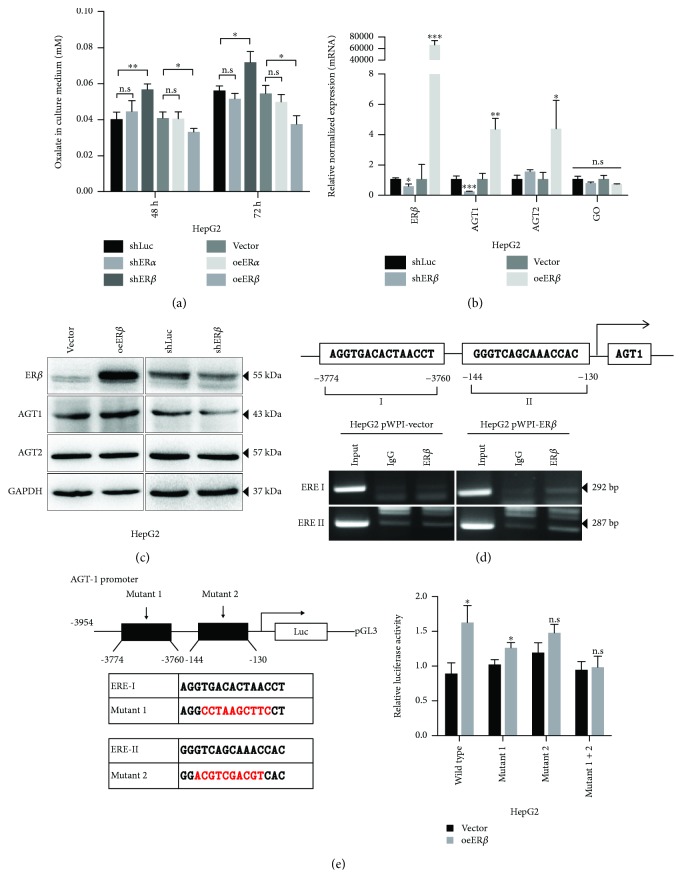
ER*β* decreases oxalate biosynthesis *via* increasing AGT1 expression in hepatocytes. (a) Oxalate level measurement in culture media of two sets of HepG2 cells: (i) shLuc, shER*α*, and shER*β* and (ii) vector, oeER*α*, and oeER*β*. sh = small hairpin RNA; oe = overexpression. (b) The mRNA expressions of the enzymes AGT1, AGT2, and GO upon manipulation of the ER*β* level in HepG2 cells. (c) Protein expressions of the enzymes AGT1 and AGT2 upon manipulation of the ER*β* level in HepG2 cells. (d) ChIP assay results in HepG2 cells showed that overexpression of ER*β* (b) increased the physical binding between ER*β* and both ERE I and ERE II. (e). Wild-type or two ERE-mutant AGT1 promoter reporter constructs were cotransfected with pRL-TK at a ratio of 1000 : 1 into HepG2-vector and HepG2-oeER*β* cells (left panel). Luciferase reporter assay was performed after 48 hr incubation (right panel). Data in (a), (b), and (e) are presented as mean ± SD, n.s: no significance. ^∗^ *P* < 0.05, ^∗∗^ *P* < 0.01, and ^∗∗∗^ *P* < 0.001.

To determine if the ER functions were involved in renal CaOx crystal deposition, we first examined the ER effects on the liver oxalate biosynthesis in the liver cells, and it was important to clarify the nonperoxisomal metabolism associated with oxalate synthesis in human hepatocytes [18]. Using the shRNA knockdown strategy, our results revealed that knockdown of the ER*β*, but not the ER*α*, led to an increased oxalate excretion (Figure 1(a)). Using the lentiviral ER-cDNA overexpression (oe) method we found that oeER*β*, but not oeER*α*, led to decreased oxalate excretion in the culture media of HepG2 cells. The above data suggests that hepatic ER*β*, and not ER*α*, might downregulate oxalate biosynthesis, which consequently led to decreased oxalate excretion.

We then examined the expression of enzymes involved in the biosynthesis of oxalate in liver and found that targeting ER*β* with ER*β*-shRNA led to little effect on the expression of GO. However, altering the ER*β via* ER*β*-shRNA or ER*β*-cDNA led to a significant alteration of both mRNA and protein expressions of AGT1, another key molecule [19] in controlling the removal of glyoxylate and prevention of its conversion to oxalate in the liver (Figures 1(b) and 1(c)).

Together, results from Figures 1(a)–1(c) suggest that ER*β* could downregulate the liver oxalate biosynthesis *via* increasing AGT1 expression.

### 2.2. Mechanism Dissection of Why ER*β* Can Increase AGT1 Expression in Hepatocytes: *via* Transcriptional Regulation

As we found ER*β* altered the AGT1 expression in both mRNA and protein levels we then assayed ER*β*'s role in regulating AGT1 at the transcriptional level. We first searched for potential estrogen response elements (EREs) on the ~3.9 kb of the AGT1 promoter region using JASPAR database and found two putative EREs located within the AGT1 promoter region (Figure 1(d), upper panel). We then applied chromatin immunoprecipitation (ChIP) *in vivo* binding assay to verify their capacity of ER*β* binding to EREs, and results revealed that ER*β* could bind to the ERE located on sites -3774 to -3760 and -144 to -130 bp away from the transcription start site of AGT1 in ER*β*-overexpressed HepG2 cells (Figure 1(d), lower panels). Importantly, ER*β* could increase the luciferase activity of the pGL3 construct of ~3.9 kb of the AGT1 promoter with wild-type ERE, but not with mutant ERE (Figure 1(e)). Together, results from Figures 1(a)–1(e) suggest that ER*β* can decrease liver oxalate biosynthesis *via* increasing the AGT1 expression at the transcriptional regulation of the AGT1 promoter.

### 2.3. ER*β* Decreases the Oxalate-Induced Oxidative Stress in the Human Renal Tubular Cells

In addition to suppressing oxalate biosynthesis in the liver, we were interested in studying ER*β*'s effects on altering the oxidative stress-induced renal cell injury as one of the potential factors impacting the renal CaOx crystal deposition.

Early studies indicated that increased renal oxalate might increase the renal injury, which might then further increase the renal CaOx crystal deposition [20, 21]. Other studies also suggested that urinary oxalate excretion in normal people without kidney stone diseases ranged from 0.31 to 0.38 mM [22], which might then increase to 0.46 to 0.53 mM in idiopathic CaOx stone patients and further increase to 0.79 to 0.90 mM in enteric hyperoxaluria patients and 0.92 to 1.43 mM in bariatric surgery patients [23]. We decided to follow previous studies [24, 25] using 0.75 mM oxalate for exploring hyperoxaluria-induced renal CaOx crystal deposition in *in vitro* renal cell studies.

**Figure 2:**
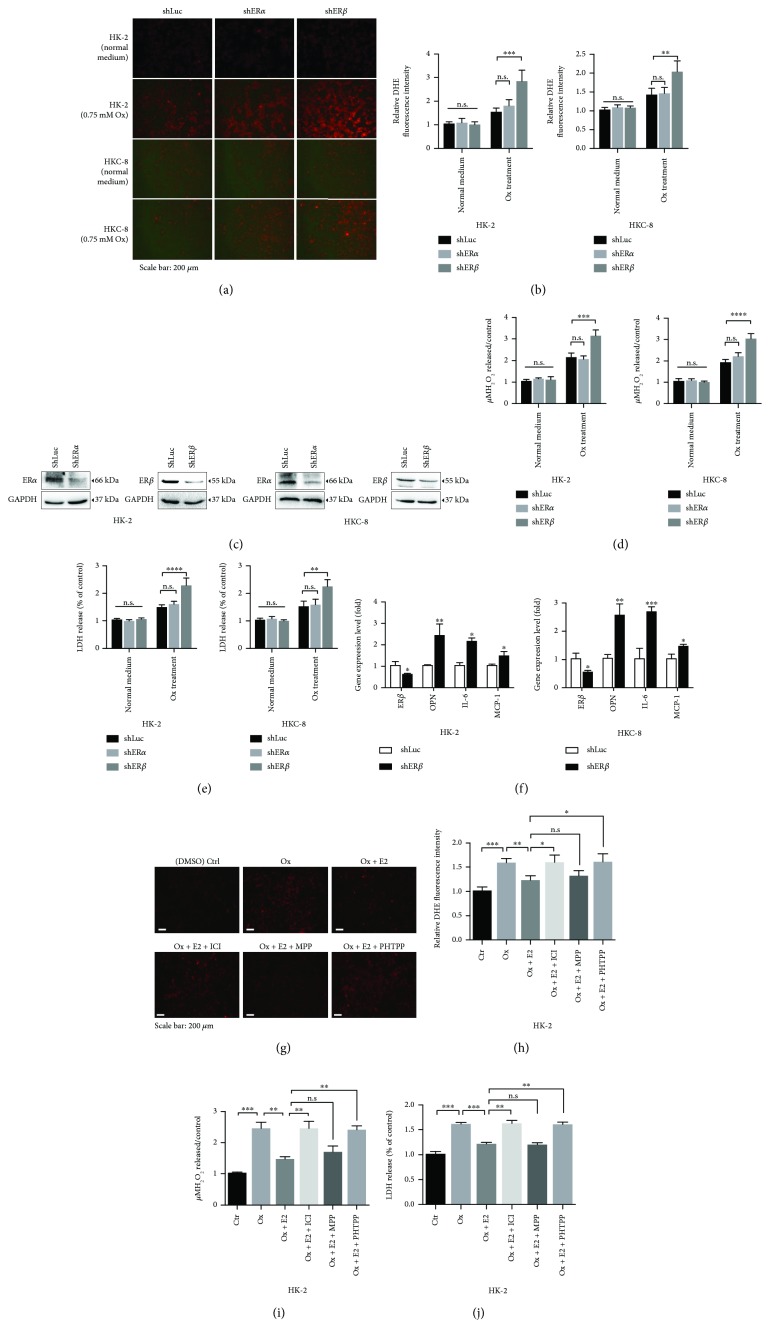
Depletion of renal ER*β* made renal epithelial cells more vulnerable to oxalate-induced ROS production and cell injury. (a) HK-2 and HKC-8 cells were transduced with lentiviral shLuc (control) or shER*α* or shER*β* and treated with normal DMEM media or DMEM media containing 0.75 mM oxalate for 6 hr, and the ROS production in the cells was detected by dihydroethidium (DHE) staining under a fluorescence microscope. The images are representative of typical staining. (b) Digital scans of DHE-stained cells were quantified using ImageJ software by comparing cells with shER*α* or shER*β* to shLuc. One-way ANOVA was applied for statistic analysis. (c) Western blots show ER*α* or ER*β* knockdown efficiency in HK-2 and HKC-8 cells. (d) Detection of H_2_O_2_ levels in culture media of the shER*α* or shER*β* or control (shLuc) renal tubular epithelial cells after challenge with 0.75 mM oxalate for 6 hr; Student's *t* tests, compared to the shLuc group. (e) LDH release measurement in the ER*α* or ER*β* knocked-down renal tubular epithelial cells treated with 0.75 mM oxalate for 6 hr. (f) The qRT-PCR analysis of inflammation-related gene expression in HK-2 and HKC-8 cells with/without sh ER*β* after 0.75 mM oxalate treatment for 6 hr. (g–j) Effect of ER antagonists on oxalate induced oxidative stress and cell injury. HK-2 cells were exposed to DMSO (Ctrl), 0.75 mM oxalate, oxalate + 10 nM E2, oxalate + 10 nM E2 + 10 *μ*M ICI, oxalate + 10 nM E2 + 10 *μ*M MPP, or oxalate + 10 nM E2 + 10 *μ*M PHTPP for 6 hr. (g) The ROS production in the cells was detected by DHE staining under a fluorescence microscope. The images are representative of typical staining. (h) Digital scans of DHE-stained cells were quantified using ImageJ software; one-way ANOVA, compared to the control group. (i) Detection of H_2_O_2_ levels in culture media of each group. (j) LDH release measurement in cells with different treatments. For (b)–(f) and (h)–(j), data are presented as mean ± SD. ns = not significant. ^∗^ *P* < 0.05, ^∗∗^ *P* < 0.01, ^∗∗∗^ *P* < 0.001, and ^∗∗∗∗^ *P* < 0.0001.

We first found that ER*β* knockdown (using shER*β*), but not ER*α* knockdown, could alter oxidative stress *via* increasing intracellular superoxide levels (Figures 2(a)–2(c)) with elevated H_2_O_2_ release (Figure 2(d)) in renal HK-2 and HKC-8 cells after exposure to 0.75 mM oxalate for 6 hr, suggesting that targeting ER*β* can alter the oxidative stress in renal cells with higher oxalate concentration.

Next, we found that knocking down ER*β*, but not ER*α*, in HK-2 cells also increased the oxidative stress-induced renal cell injury (cytotoxicity) *via* measuring LDH activity after exposure to 0.75 mM oxalate for 6 hr (Figure 2(e)). Similar results were also obtained when we used the 2nd ER*β*-shRNA (Supplementary Figure 1).

Importantly, we also that found knockdown of ER*β* in HK-2 and HKC-8 cells led to an increased expression of oxidative stress/inflammation-related genes, including osteopontin (OPN), interleukin-6 (IL-6), and monocyte chemotactic protein 1 (MCP-1), at mRNA levels after exposure to 0.75 mM oxalate for 6 hr (Figure 2(f)).

Furthermore, we tested 3 different ER antagonists: (i) ICI 182,780 is an antagonist for both ER*α* and ER*β*, (ii) 4-[2-phenyl-5,7-bis(trifluoromethyl)pyrazolo(1,5-a)pyrimidin-3-yl]phenol (PHTPP) is a ER*β*-selective antagonist, and (iii) methylpiperidino pyrazole (MPP) is a selective ER*α* antagonist. HK-2 cells were cultured under media with CS-FBS for 48 hr; as expected, 10 nM E2 treatment could downregulate oxalate-mediated oxidative stress and cell injury via reducing the superoxide production. When HK-2 cells were further treated with 10 *μ*M antagonists, we found that ICI 182,780 or ER*β* special antagonist PHTPP, but not the ER*α* antagonist MPP, could significantly increase oxalate-mediated oxidative stress and cell injury *via* inducing the superoxide production (Figures 2(g)–2(j)).

Together, results from Figures 2(a)–2(j) demonstrate that targeting E2/ER*β* signaling *via* (i) antiestrogens to suppress E2-activated ER*β* or (ii) knocking down ER*β* by shRNA to suppress the ER*β* all led to increasing oxidative stress-induced cell injury in human renal epithelial cells (with 0.75 mM oxalate treatment), suggesting that activation of ER*β* functions may protect renal tubular cells against oxidative stress-induced renal injury in patients with hyperoxaluria.

### 2.4. Mechanism Dissection of Why ER*β* Suppresses the Oxidative Stress-Induced Renal Cell Injury: *via* Suppressing the NADPH Oxidase Subunit NOX2 Expression

**Figure 3:**
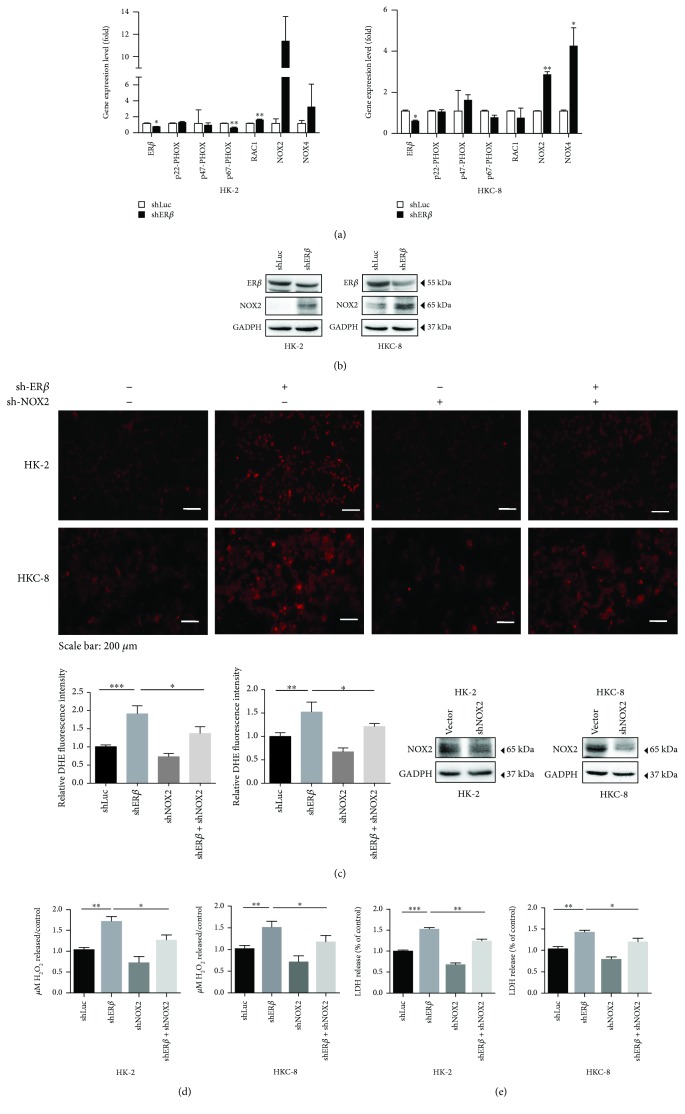
ER*β* inhibits oxalate-induced oxidative stress *via* modulating NOX2 expression. (a) The qRT-PCR analysis of NADPH oxidase subunits after knocking down ER*β* in HK-2 and HKC-8 cells. Cells were transduced with shER*β* or with shLuc as control after 72 hr incubation, and then treated with 0.75 mM oxalate for 6 hr. (b) Western blot analysis of NOX2 expression in renal cells with/without shER*β* after 6 hr exposure to 0.75 mM oxalate. (c) Rescue assay using HK-2 and HKC-8 cells with/without shER*β*. shNOX2 showed partially reversed knockdown of ER*β*- (shER*β*-) induced ROS production. All cells were exposed to 0.75 mM oxalate for 6 hr prior to collection for data analysis. Upper panels show representative images of DHE staining, and quantification is shown in the lower left panel. Western blot in the lower right panel shows NOX2 knockdown efficiency. (d) After treating with 0.75 mM oxalate for 6 hr, shNOX2 can partly reverse the silenced ER*β*-induced H_2_O_2_ production in conditioned media. (e) Rescue assay using HK-2 and HKC-8 cells with/wthout shNOX2 showed partially reversed shER*β*-induced LDH release. All the cells in Figures 3(a)–3(e) were exposed to 0.75 mM oxalate for 6 hr prior to collection for data analysis. For (a), (c), (d), and (e), data are presented as mean ± SD. ^∗^ *P* < 0.05, ^∗∗^ *P* < 0.01, and ^∗∗∗^ *P* < 0.001.

**Figure 4:**
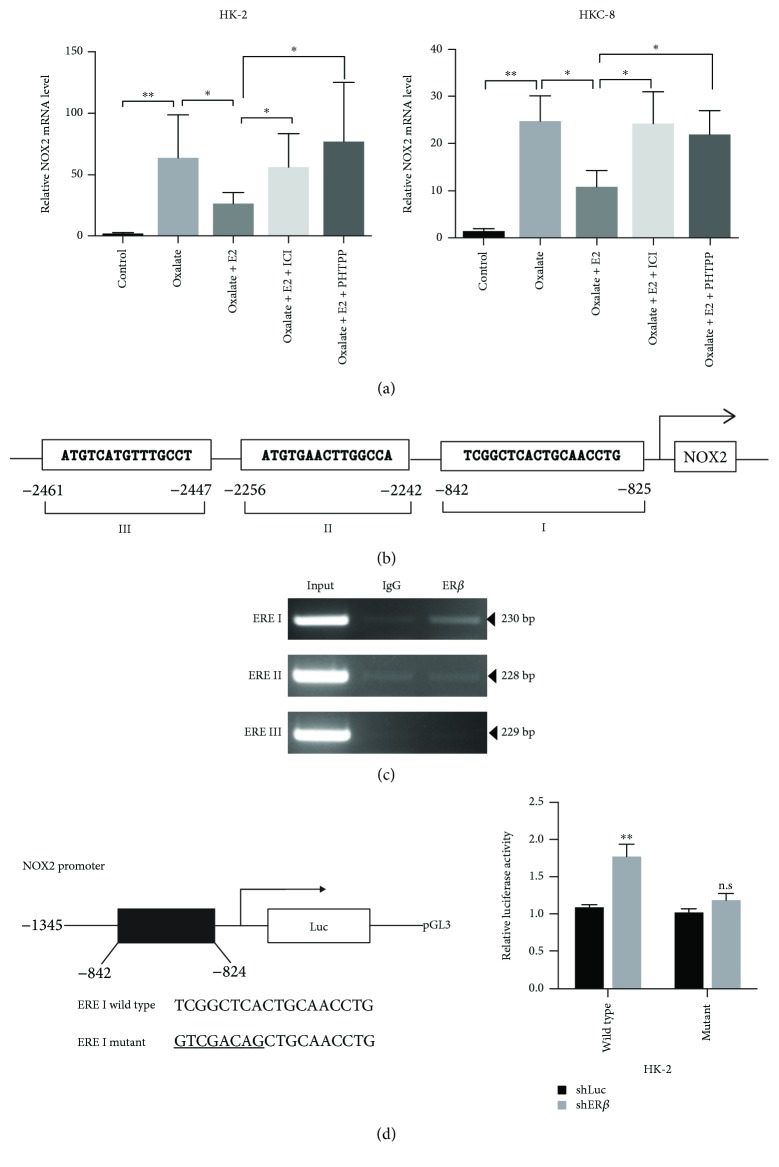
Mechanism dissection on how ER*β* regulates NOX2 expression. (a) NOX2 mRNA levels were regulated by E2 or ER*β* antagonists in HK-2 and HKC-8 cells. Cells were treated for 6 hr with vehicle (control), 0.75 mM oxalate, 0.75 mM oxalate + 10 nM E2, 0.75 mM oxalate + 10 nM E2 + 10 *μ*M ICI, or 0.75 mM oxalate + 10 nM E2 + 10 *μ*M PHTPP for q-PCR of NOX2 mRNA levels. Bonferroni's test was applied for statistical analysis of results. (b) Predicted 3 potential EREs located in the 3 kb-NOX2 promoter region. (c) ChIP was performed in HK-2 cells, and only ERE I of the NOX2 promoter could be bound by ER*β*. (d) ERE I wild-type or mutant of NOX2 promoter reporter constructs were cotransfected with pRL-TK at a ratio of 1000 : 1 into HK-2-shLuc and HK-2-shER*β* cells. Luciferase reporter assay was performed after 48 hr incubation. For (a) and (d), data shown are mean ± SD. ^∗^ *P* < 0.05 and ^∗∗^ *P* < 0.01. n.s. = not significant.

To dissect the mechanism(s) of how ER*β* can decrease the oxalate-mediated oxidative stress-induced renal cell injury, we first focused on NADPH oxidases, the key components related to ROS in renal tubular epithelial cells [26], including NOX1-5, p22-phox, p47-phox, p67-phox, and Rac1 [27]. Among those genes, NOX2 expression is upregulated in both cell lines, yet NOX4 expression is increased in only the HKC-8 cells; therefore, we choose NOX2 for further functional studies. Using qRT-PCR and western blot assays, we found that knocking down ER*β* in HK-2 or HKC-8 cells led to an increase in the expression of NOX2 at mRNA and protein levels after exposure to 0.75 mM oxalate for 6 hr (Figures 3(a) and 3(b), respectively). Consistent results were also obtained when we replaced ER*β*-shRNA with ER antagonist ICI or ER*β* special antagonist PHTPP (see Figure 4(a)).

We then applied an interruption approach using NOX2-shRNA to prove that ER*β* could function *via* decreasing NOX2 expression to inhibit oxalate-mediated, oxidative stress-induced, renal injury. As expected, the results from 3 different assays including dihydroethidium (DHE) staining (Figure 3(c)), H_2_O_2_ release assay (Figure 3(d)), and LDH assay (Figure 3(e)) all revealed that knocking down NOX2 with NOX2-shRNA led to partial reversal of the ER*β*-shRNA-increased, oxalate-mediated, oxidative stress-induced cell injury in the renal HK-2 and HKC-8 cells.

Together, results from Figures 3(a)–3(e) suggest that ER*β* suppresses the oxalate-mediated oxidative stress-induced renal cell injury *via* suppressing the NOX2 expression.

### 2.5. Mechanism Dissection of How ER*β* Suppresses NOX2 Expression: *via* Transcriptional Regulation

To further dissect the molecular mechanism of how ER*β* regulates NOX2 expression in renal tubular epithelial cells, we first noticed that the NOX2 mRNA expressions were increased in both HK-2 and HKC-8 cells after 6 hr of 0.75 mM oxalate exposure (Figure 4(a)). As expected, treating with 10 nM E2 prevented oxalate-induced NOX2, and this reduction was reversed after treating with nonselective ER antagonist ICI or selective ER*β* antagonist PHTPP, suggesting that a transcriptional mechanism may contribute to NOX2 downregulation by ER*β* (Figure 4(a)).

We then searched for potential EREs on the 3 kb of the NOX2 promoter region using the JASPAR database, and results revealed three putative EREs located within the NOX2 promoter region (Figure 4(b)). We then performed the ChIP *in vivo* binding assay, and results revealed that ER*β* could bind to ERE-I located -842 to -825 bp upstream of the transcriptional start site of NOX2 in HK-2 cells (Figure 4(c)). Furthermore, using the luciferase assay to examine 1.3 kb of the NOX2 promoter region that was linked to the pGL3-luciferase reporter plasmid, we found that knocking down ER*β* with shRNA (shER*β*) in the HK-2 cells could increase the luciferase expression of wild-type NOX2 promoter construct, but not the NOX2 promoter construct with mutated ERE-1 (Figure 4(d)).

Together, results from Figures 4(a)–4(d) suggest that ER*β* can suppress NOX2 expression at the transcriptional level *via* binding to the ERE located in its 5′ promoter region.

### 2.6. Using *In Vivo* Mouse Models with PHTPP Injection to Increase the Renal CaOx Crystal Deposition

**Figure 5:**
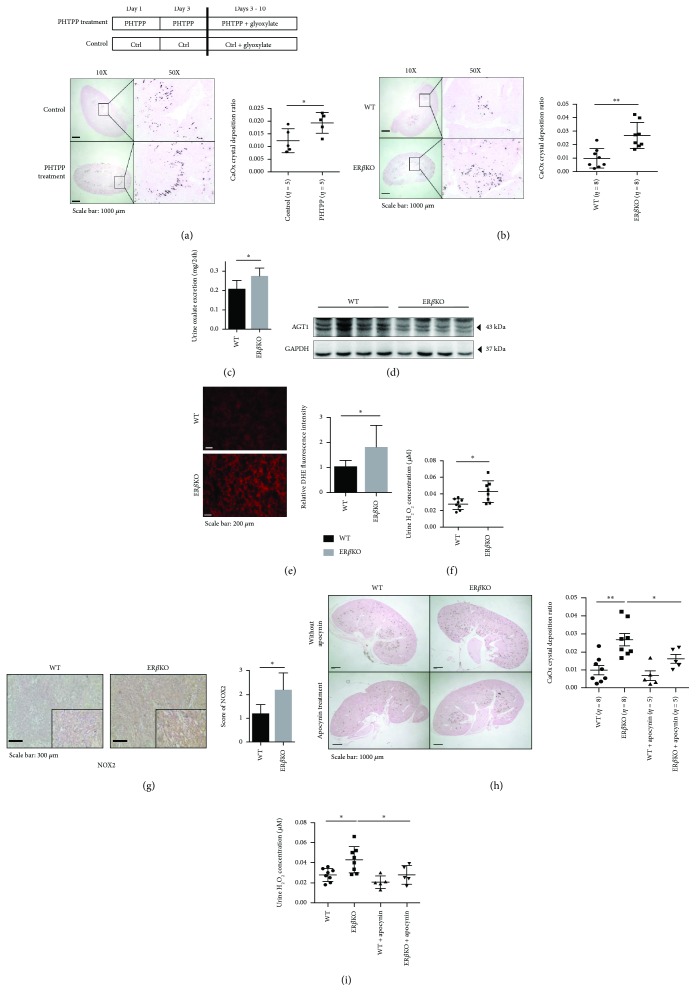
*In vivo* mouse model data confirm that ER*β* deficiency promotes renal CaOx crystal deposition *via* increasing the hepatic oxalate biosynthesis and renal oxidative stress. (a) Diagram describing the injection schedule for mock control, a selective ER*β* antagonist PHTPP, and glyoxylate (upper panel). Crystal staining (lower panels) showing the deposition of CaOx crystals in kidney tissues of the PHTPP-treated and mock-control mice. CaOx crystal formation was detected by Pizzolato staining. Crystallization in each kidney section was quantified by calculating the ratio (percent) of the area containing crystals to the entire kidney section using ImageJ software. (b) Staining (left panels) and quantification (right panels) results for CaOx crystal deposition as detected by Pizzolato staining in kidney tissues of the ER*β*KO and their WT female littermate mice. (c) Detection of 24 hr oxalate excretion in urine samples collected from the ER*β*KO and WT female mice prior to sacrifice. (d) Western blot of 60 *μ*g liver protein from ER*β*KO and WT mice probed with affinity-purified rabbit antibody raised against recombinant mouse AGT1 shows lower expression of AGT1 in the ER*β*KO mice. (e) Fresh-frozen renal tissues were stained for 30 min with DHE. Representative sections from the ER*β*KO and their WT female littermate mice are shown (left panels). Right bar graphs show quantification of superoxide generation. (f) H_2_O_2_ concentration in urine sample from ER*β*KO and WT female mice. (g) Representative micrographs of NOX2 immunostaining in renal tissue from the ER*β*KO and their WT littermate mice. The NOX2 protein was mainly located in renal tubular cells. Quantification of NOX2 is shown in the right panel. Scores were classified as 0 to 3, based on the intensity of staining and the percentage of positive cells. (h) Inhibitory effect of apocynin treatment in mice. CaOx crystal formation in ER*β*KO mice was significantly decreased after *in vivo* apocynin treatment. (i) Apocynin treatment reduced H_2_O_2_ concentration in urine samples from ER*β*KO mice. For (a)–(c) and (e)–(i), data are presented as mean ± SD. ^∗^ *P* < 0.05 and ^∗∗^ *P* < 0.01.

To confirm all above *in vitro* cell line results with the *in vivo* mouse model, we first investigated whether PHTPP, a ER*β* selective antagonist, could promote renal CaOx crystal deposition in a mouse model. C57BL/6J female mice were treated with DMSO or PHTPP (10 *μ*l of 1 × 10^−2^ M PHTPP per mouse) *via* intraperitoneal injection on days 1, 3, 5, 7, and 9. In addition, all mice were given daily intraperitoneal injections of glyoxalate (80 mg/kg/day) from days 3 to 10 to induce renal CaOx crystal deposition (Figure 5(a), upper panel) [28]. The results revealed that mice with ER*β* selective antagonist PHTPP treatment had more renal CaOx crystal deposition compared to controls (Figure 5(a), lower panel).

### 2.7. Using *In Vivo* ERKO Mouse Models Showing Deletion of ER*β* Gene Led to Increase in Renal CaOx Crystal Deposition

We then applied the 2nd mouse model using ER*β* knockout (ER*β*KO) mice *via* breeding heterozygous ER*β*KO (ER*β* ^+/-^) male and female mice to generate the homozygote ER*β*KO (ER*β* ^−/−^) mice that lack both copies of the ER*β* genes (see Supplementary Figure 2 for breeding and genotyping of the homozygote ER*β*KO mice). These ER*β*KO mice, plus their control wild-type (WT) mice, were given daily intraperitoneal injections of glyoxalate (80 mg/kg/day) for 7 days to induce intrarenal CaOx crystal deposition in the parenchyma. The results from Pizzolato staining revealed that ER*β*KO mice had more renal CaOx crystal deposition than the WT littermate mice (Figure 5(b)).

Metabolism cages were used for control of diet and excretion of each mouse. One day before sacrifice, we collected urine samples and examined the 24 hr urinary oxalate excretion in ER*β*KO and WT mice and found that ER*β*KO mice had higher urinary oxalate than the WT littermate controls (Figure 5(c)). Consistently, we found that AGT1 expression was lower in the livers collected from ER*β*KO mice than in WT mice after sacrifice (Figure 5(d)).

We then examined oxidative stress *via* measuring the ROS production in these mouse kidneys using the reporter molecule DHE, and results revealed that the kidney sections of ER*β*KO mice had significantly stronger nuclear fluorescent signals as compared with those of WT mice (Figure 5(e)). As expected, prior to sacrifice, we found a much higher H_2_O_2_ concentration in the urine of ER*β*KO mice than found in WT mice (Figure 5(f)), and results from the NOX2 IHC data were scored and results showed a higher NOX2 expression in renal tubular cells of ER*β*KO mice compared to WT mice (Figure 5(g)).

Taken together, the results from two different mouse models confirm the *in vitro* cell line data showing that targeting E2/ER*β* signaling with either antiestrogen (PHTPP) or ER*β* (knockout gene) all lead to increased renal CaOx crystal deposition with increased urinary oxalate *via* altering the liver oxalate biosynthesis and renal oxidative stress-induced renal cell injury.

### 2.8. Using *In Vivo* ERKO Mouse Models to Prove the Treatment with NADPH Oxidase Inhibitor Apocynin Can Suppress the CaOx Crystal Deposition on the Parenchyma

Then, to connect all above *in vitro/in vivo* studies to potential future clinical applications, we applied the ER*β*KO mouse model and treated the mice with the small molecule NADPH oxidase inhibitor, apocynin [29–31]. Results revealed that injecting apocynin could partly reverse the ER*β*KO-increased renal CaOx crystal deposition (Figure 5(h)) and oxidative stress (*via* measuring the level of H_2_O_2_ in urine) (Figure 5(i)), suggesting that targeting the ER*β*-regulated NOX2 expression with apocynin may present a potential novel therapy to suppress renal CaOx crystal deposition.

### 2.9. Using *In Vivo* Rat Models to Confirm ER*β* Can Decrease the Intrarenal CaOx Crystal Deposition on the Parenchyma

To overcome the potential glyoxalate side effects to damage the kidney in the mouse [32], we also applied the 4th animal model using rats fed with hydroxy-L-proline (HLP) for 8 weeks to induce CaOx crystal deposition, since early studies indicated that this rat model has little acute renal injury and more closely mimicks the renal CaOx crystal deposition occurring in human kidney stone formers [33, 34].

**Figure 6:**
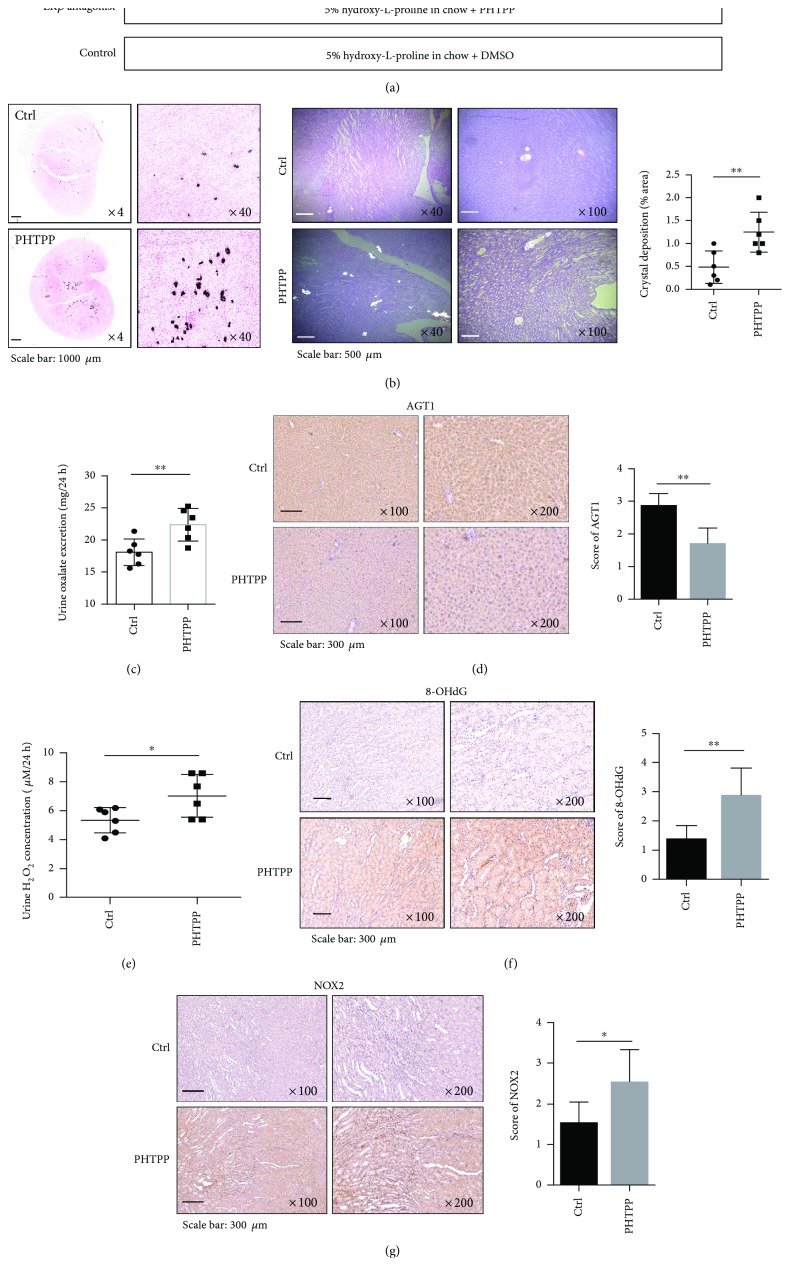
Suppressing ER*β* activity could increase renal CaOx crystal formation in HLP-induced rat model. (a) A diagram describing the injection schedule for PHTPP/DMSO treatment. (b) Crystal staining showing the deposition area of CaOx crystals in kidney tissues of the rats treated with PHTPP or vehicle control. CaOx crystal formation was detected by Pizzolato staining and imaged by polarized light optical microphotography. (c) The PHTPP-treated rats showed increased urine oxalate excretion compared to vehicle-treated control rats. Prior to sacrifice, we collected and detected 24 hr oxalate excretion in urine of rats treated with PHTPP or vehicle control. (d) IHC staining of AGT1 in liver tissues of the rats treated with PHTPP or vehicle. The PHTPP-treated rats showed decreased AGT1 expression. (e) Detection of 24 hr urine H_2_O_2_ levels in the rats treated with PHTPP or vehicle. The PHTPP-treated rats showed higher H_2_O_2_ levels. (f) IHC staining of 8-OHdG in kidney tissues of the rats treated with PHTPP or vehicle. The PHTPP-treated rats showed increased 8-OHdG expression. (g) IHC staining of NOX2 in kidney tissues of the rats treated with PHTPP or vehicle. The PHTPP-treated rats showed increased NOX2 expression as compared to control rats. For (b)–(g), data are presented as mean ± SD. ^∗^ *P* < 0.05 and ^∗∗^ *P* < 0.01.

Eight-week-old female rats were fed with chow mixed with 5% HLP (weight/weight HLP/chow) to induce the hyperoxaluria and CaOx crystal deposition (Figure 6(a)) and then treated with PHTPP (850 *μ*g/kg per rat) or DMSO mock control *via* intraperitoneal injection every other day for 8 weeks. Kidneys were collected for examining the renal CaOx crystal deposition and results revealed that little renal CaOx crystal deposition was found in the control DMSO-treated group. In contrast, renal CaOx crystal deposition was prevalent in rats treated with PHTPP (Figure 6(b)).

Prior to sacrifice, 24 hr urine samples were collected from each mouse (using metabolism cages). Results from the 24 hr urinary oxalate assay also revealed that rats treated with PHTPP have significantly higher urinary oxalate than the controls (Figure 6(c)). In addition, there was lower expression of AGT1 in the liver of rats treated with PHTPP (Figure 6(d)).

We also examined the ROS production by detecting H_2_O_2_ concentrations in the urine samples and found a much higher H_2_O_2_ concentration in the urine of rats treated with PHTPP than in urine of the control rats (Figure 6(e)). The consequences of the ER*β*-suppressed, oxalate-mediated, oxidative stress-induced renal injury were also demonstrated by the increased 8-hydroxy-2′-deoxyguanosine (8-OHdG) levels, a critical biomarker of oxidative stress [15], in the kidney tissues of rats in the PHTPP group compared with the control group (Figure 6(f)). IHC staining also showed a higher NOX2 expression in renal tubular cells of rats treated with PHTPP as compared with the control rats (Figure 6(g)).

Taken together, the results from HLP-induced CaOx crystal deposition rat model (Figures 6(a)–6(g)) are in agreement with all above *in vitro* cell lines and *in vivo* mouse model studies showing that ER*β* can suppress the renal CaOx crystal deposition *via* altering the liver oxalate biosynthesis and inhibiting renal oxidative stress-induced cell injury.

## 3. Discussion

CaOx crystal formation is one of the metabolic disorders in which multiple factors, such as obesity, hypertension, and diabetes, might be involved. Furthermore, dysfunctions of the gut, liver, and kidney are related to CaOx crystal formation [15].

**Figure 7:**
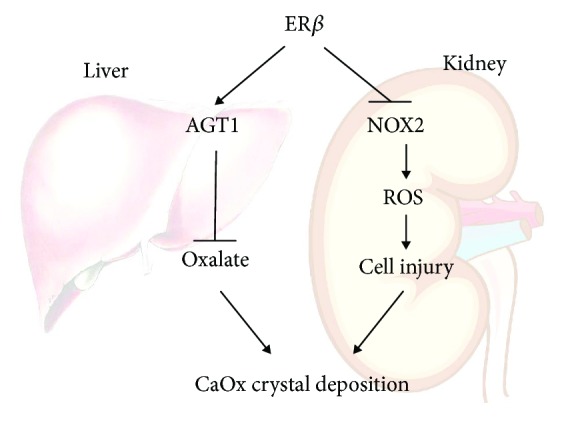
Scheme. The schematic illustration of the ER*β* signal regulation of CaOx crystal formation in the liver and kidney. For the liver part, ER*β* signaling promotes activity of AGT1, which decreases the biosynthesis of oxalate by converting glyoxylate into glycine decreasing CaOx crystal formation. For the kidney part, ER*β* has a protective effect in human renal tubular cells against oxalate-induced oxidative stress *via* suppression of NOX2 (a NADPH oxidase), which finally leads to decreasing CaOx crystal deposition on the damaged cell surface.

Hyperoxaluria is a key risk factor for the development of kidney stones [35], and urinary oxalate excretion is a biomarker for the development of kidney stones [36–38]. Reduction of urinary oxalate is associated with a lower recurrent rate of CaOx kidney stones [39]. Urinary oxalate is derived from two major sources, 80% to 90% comes from endogenous production in the liver with the rest obtained from dietary oxalate [40, 41]. In the liver, AGT1 is a key enzyme in glyoxylate detoxification [19] and is necessary to avoid oxalate formation from glyoxylate. Under normal circumstances, AGT1 metabolizes oxalate precursors into the harmless amino acid glycine, which is then used by the body or is excreted. When AGT1 expression or function decreases, oxalate production is elevated in the liver, consequently resulting in an increased renal CaOx crystal deposition [19]. Primary hyperoxaluria type 1, a rare inherited metabolic disorder, results from a deficiency of AGT1 and leads to increased oxalate synthesis. In the present study, our findings indicate that ER*β* could enhance hepatic AGT1 expression *via* direct binding to the AGT1 gene 5′ promoter to decrease endogenous oxalate production, leading to reduction in urinary oxalate excretion, and then further suppress the renal CaOx crystal deposition (Figure 7).

Oxidative stress is the condition in which the production of ROS is greater than the protective capacity of antioxidant enzymes, such as superoxide dismutase. The role of oxidative stress in kidney stone formation has received increasing attention in recent years [17, 26, 42, 43]. Increasing evidence suggested that oxidative stress-induced renal injury might play key roles to promote the renal CaOx crystal deposition under higher urinary oxalate concentrations, a condition seen in patients with hyperoxaluria [44], which may involve inducing cell apoptosis through mitochondrial destruction with microvilli being injured and disintegrated. These resulting materials may then appear in the renal tubular lumen and become part of the nuclei of Ca-phosphate (CaP) that is gradually wrapped/coated by CaOx (under hyperoxaluria) and some inflammatory or fibrotic proteins, for example OPN, to finally become the major renal crystal matrix. The movement of these renal CaP-CaOx crystal matrices into the kidney tubular lumen may then cause some mechanical injuries leading to local bleeding. Consequences of these renal injuries may then induce these crystal matrices to become exposed/attached to the renal parenchyma [17, 43]. Another report demonstrated that antioxidant treatment can reduce renal CaOx crystal deposition *in vivo* [45].

There are at least three distinct ERs expressed in the kidney. Two ERs, ER*α* and ER*β*, belong to the steroid hormone receptor superfamily and function as ligand-activated transcription factors. The third one, GPR30, has been recently studied and belongs to the G-protein-coupled receptor superfamily and may promote nongenomic signaling events by estrogen. The potential role of E2 in regulating renal function is evident from the observation that the kidney expresses the classical ER*α* and ER*β*. In human fetal kidneys, ER*β* is the prominent renal expressed ER, whereas ER*α* is only marginally expressed [46]. Recent studies also found that ERs (ER*α* or ER*β*) might play protective roles against oxidative stress-induced cell injury in several tissues, including the brain [12], skin [47], kidney [48–50], and myocardium [51]. However, the mechanisms by which ERs can suppress the oxidative stress-induced renal injury in the presence of oxalate remain unclear [49]. Because ER*α* and ER*β* can respond differently to E2 in mediating its transcriptional activity, it is possible that the differential expression of ER subtypes may be of physiological relevance. Kim et al. [52] *r*eported that ER*α* function is involved in the regulation of renal fibroblast activation *via* the TGF-*β*1/Smad signaling pathway. Aufhauser et al. [53] found that ER*α* mediated female protection from renal ischemia-reperfusion injury through mechanisms extrinsic to the kidney. Interestingly and importantly, the current study provides evidence that ER*β*, but not ER*α*, can protect against oxidative stress-induced renal injury in the presence of oxalate, which may then lead to suppressing the renal CaOx crystal deposition.

NADPH oxidase is a major source of ROS in the kidneys [54]. NOX-derived ROS are implicated in physiological processes of the kidney, including gluconeogenesis, glucose transport, tubuloglomerular feedback, hemodynamics, and electrolyte transport. NOX2 is the first described NADPH oxidase with a sole function of producing ROS. Of the seven NOX isoforms, NOX2 might be the best characterized in renal pathology. We identified NOX2, a NADPH oxidase, as a source of ROS induced by oxalate. Increased oxalate concentration could dramatically stimulate NOX2 expression in renal cells within a short time, and NOX2 depletion not only diminished knockdown of ER*β*-induced ROS formation but also reduced cell injury. This response was confirmed *in vivo* in mice treated with apocynin, a NADPH oxidase inhibitor, showing diminished ROS production and crystal formation in ER*β*KO mouse kidneys. Our results confirmed that ER*β* protects the kidney against oxidative stress-induced renal injury *via* suppressing the NOX2 expression (Figure 7). This finding also points to a novel strategy *via* targeting NOX2 to reduce the renal CaOx crystal deposition in the presence of oxalate, a condition seen in patients with hyperoxaluria.

In summary, this study is the first report showing that E2/ER*β* signaling suppresses kidney CaOx crystal deposition *via* reducing hepatic biosynthesis of oxalate and inhibiting oxidative stress-induced renal injury.

## 4. Materials and Methods

### 4.1. Cell Lines

The human HK-2, HepG2, and HEK-293 cell lines were purchased from the American Type Culture Collection (ATCC) (Rockville, MD). The HKC-8 cell line was kindly provided by Dr. Syed Khundmiri of the University of Louisville (Louisville, Kentucky). All the cells were maintained in DMEM media with 8% fetal bovine serum and 1% penicillin/streptomycin.

### 4.2. Reagents and Materials

GAPDH (6c5) and ER*α* (MC-20) antibodies were purchased from Santa Cruz Biotechnology (Santa Cruz, CA). ER*β* (N2C2) antibody was purchased from GeneTex (Irvine, CA). NOX2 antibody was purchased from BosterBio (PA1667, Pleasanton, CA). Anti-mouse/rabbit second antibody for Western Blot was from Invitrogen (Grand Island, NY). Normal rabbit IgG was also from Santa Cruz Biotechnology.

### 4.3. Lentiviral Transduction of Targeted Mammalian Cells

The pLVTHM-shNOX2, pLKO.1-shER*α*, pLKO.1-shER*β*, pLKO.1-shLuc, pWPI-vector, pWPI-ER*α*, or pWPI-ER*β* as well as the psPAX2 packaging plasmid and pMD2G envelope plasmid were transfected into HEK-293 cells using the standard calcium chloride transfection method for 48 hr to get the lentivirus soups. The lentivirus soups were collected and concentrated by density gradient centrifugation. The collected virus were added to the target cells in the presence of polybrene (2 *μ*g/ml) to incubate for 24 hr or frozen at -80°C for later use. Cells were refreshed with culture media and cultured for another 3 days to allow target protein expression.

### 4.4. Oxalate Treatments and ROS Detections Using Hydrogen Peroxide Assay and DHE Staining

Sodium oxalate (Sigma) stock solution (10 mM) in PBS was diluted in media to achieve a final concentration of 0.75 mM. HK-2 or HKC-8 cells with/without knockdown of ER*α* or ER*β* were seeded in 12-well plates (20,000 cells/1 ml regular media per well) incubated overnight. The next day, the cell media were changed to media with 0.75 mM oxalate, and after 6 hr we then collected the cultured media to detect H_2_O_2_ amounts. The H_2_O_2_ concentration in the media, or in the mouse urine samples, was detected using Sigma Fluorimetric Hydrogen Peroxide Assay Kit (MAK165) following the manufacturer's protocol. Dihydroethidium (DHE) (Cayman, No. 12013) was diluted in PBS to the final concentration of 10 *μ*M, and 200 *μ*l of DHE-PBS solution was added onto cell monolayers or frozen renal tissue sections (5 *μ*m) by dropping, and the reaction was incubated in the dark at 37°C for 30 min. Excess DHE was washed away by 1x PBS twice, and cells/tissues on the slides were fixed and stained. Images were captured immediately with a fluorescent microscope at excitation and emission wavelengths of 520 and 610 nm, respectively. For quantitative assessments, the images were analyzed by using ImageJ software.

### 4.5. Oxalate Measurement

Oxalate measurements in the HepG2 cell culture media and 24 hr mouse/rat urine collections were determined using an Oxalate Kit (Trinity Biotech) according to the manufacturer's instructions. The oxalate concentration in cells was measured by ion chromatography following Baker et al.'s procedure [18]. Briefly, cell monolayers were washed three times by PBS and extracted with ice-cold 10% trichloroacetic acid (TCA) for 30 min. The TCA was removed from extracts by vigorously vortexing with an equal volume of 1,1,2-trichlorotrifluoroethane (Freon)-trioctylamine (3 : 1, vol/vol), centrifuging at 4°C to promote phase separation, and collecting the upper aqueous layer for analysis.

### 4.6. Lactate Dehydrogenase (LDH) Release Assay (Cytotoxicity Assay)

After cells were seeded in 96-well plates at the density of 10^4^ cells/well and cultured overnight, the confluent monolayers of cells were treated with DMEM containing 0.75 mM oxalate for 6 hr. After centrifugation, the LDH amounts in supernatants were determined according to the manufacturer's instructions (Thermo Fisher Scientific, Rochester, NY). The optical products were read at 490 nm. Values were normalized to those for control shLuc group samples.

### 4.7. RNA Extraction and Quantitative Real-Time PCR Analysis

Total RNA was extracted by TRIzol reagent (Invitrogen) according to the manufacturer's instructions. RNAs (1 *μ*g) were subjected to reverse transcription using Superscript III transcriptase (Invitrogen). Quantitative real-time PCR (qRT-PCR) was conducted using a Bio-Rad CFX96 system with SYBR Green to determine the mRNA expression level of a gene of interest. RNA expression levels were normalized to the expression of GAPDH. The sequences of primers used are in Supplementary Table 1.

### 4.8. Western Blot Assay

Total protein was extracted by RIPA buffer containing 1% protease inhibitors (Amresco, Cochran, CA). Proteins (30-50 *μ*g) were separated on 10% SDS/PAGE gel and then transferred onto PVDF membranes (Millipore). After blocking the membranes, they were incubated with appropriate dilutions (1 : 1000) of specific primary antibodies, and the immunopositive bands were visualized with an ECL chemiluminescent detection system (Thermo Scientific).

### 4.9. Chromatin Immunoprecipitation Assay (ChIP)

Cell lysates were precleared sequentially with normal rabbit IgG and protein A-agarose. Anti-ER*β* antibody (5.0 *μ*g) was added to the cell lysates and incubated at 4°C overnight. For the negative control, IgG was used in the reaction. Specific primer sets were designed to amplify a target sequence within human AGT1 or NOX2 promoters, and PCR products were identified by agarose gel electrophoresis.

### 4.10. Luciferase Reporter Assays

For luciferase assays, 3954 bp fragments of AGT1 promoters or 1345 bp fragments of NOX2 promoters containing wild-type or mutant ERE-binding sites were constructed into the pGL3 basic vector (Promega). Cells were plated in 24-well plates and transfected with the above promoter luciferase pGL3 plasmid and pRL-TK luciferase plasmid using Lipofectamine 3000 (Invitrogen) according to the manufacturer's instructions. After the indicated treatments, cells were lysed and the luciferase activity was detected by the dual-luciferase Assay (Promega).

### 4.11. Breeding ER*β*KO Mice and Development of the CaOx Crystal Mouse Model

Animals were housed in a vivarium at the University of Rochester Medical Center, School of Medicine and Dentistry. All protocols related to animals were overseen and approved by the University Committee for Animal Research, and all animals were treated in accordance with National Institutes of Health guidelines.

The ER*β* knockout (ER*β*KO, 129P2-Esr2^tm1Unc^/J) and the background wild-type (WT) mice were purchased from the Jackson Laboratory (Bar Harbor, ME) and bred to heterozygote ER*β*KO (ER*β* ^+/-^) male and female mice. And the ER*β* ^+/-^ male and ER*β* ^−/−^ female mice were mated to obtain homozygote ER*β*KO (ER*β* ^−/−^) female mice for experiments. The genotypes of the female pups were confirmed by polymerase chain reaction (PCR) [55].

We established the CaOx crystal mouse model following the reported protocol [28]. The 8-week-old female mice were given daily intraperitoneal injections of 80 mg/kg glyoxylate (G1134, Sigma, St. Louis, MO) for 7 days. All animals had free access to chow and water. The 24 hr urine samples were collected 1 day before sacrifice using metabolism cages to control the diet and excretion of each animal, and mouse liver and kidney tissues were collected after sacrifice. As we are testing the ER*β* gene knockout effects in our animal models, those mice were not ovariectomized.

For the *in vivo* rescue experiment, apocynin (10 mg/kg/day) or saline was injected ip into groups of mice every day for 7 days, followed by coinjection with glyoxylate (80 mg/kg/day) for 7 days. After treatments, the mice were sacrificed and kidneys were obtained for further analysis.

### 4.12. Development of Hyperoxaluria-Induced CaOx Crystal Formation in Rats and Test the Effects of ER*β* Antagonist PHTPP

Eight-week-old Sprague-Dawley female rats, from the Guangdong Medical Laboratory Animal Center, were divided into 2 groups (6 rats/group), and both were given chow mixed with 5% HLP (weight/weight HLP/chow) under either DMSO or PHTPP (850 *μ*g/kg per rat) intraperitoneal injection every other day for a total of 8 weeks. As we are testing the antiestrogen effects in our animal model, those female rats were not ovariectomized. The 24 hr urine samples were collected 1 day before sacrifice, and rat liver and kidney tissues were collected after sacrifice.

### 4.13. H&E and Immunohistochemical (IHC) Staining

Tissues were fixed in 10% (*v*/*v*) formaldehyde in PBS, embedded in paraffin, and cut into 5 *μ*m sections and used for H&E staining and IHC staining with specific primary antibodies against AGT1, NOX2, and 8-OHdG. To enhance antigen exposure, the slides were treated with 10 mM sodium citrate (pH = 6.0) at 98°C for 10 minutes for antigen retrieval. The slides were incubated with endogenous peroxidase blocking solution and then were incubated with the primary antibody at 4°C overnight. After rinsing with PBS, the slides were incubated for 45 minutes with biotin-conjugated secondary antibody, washed, and then incubated with enzyme conjugate horseradish peroxidase (HRP) streptavidin. Freshly prepared DAB (Zymed, South San Francisco, CA) was used as substrate to detect HRP. Finally, slides were counterstained with hematoxylin and mounted with aqueous mounting media. Positive cells were calculated as the number of immunopositive cells × 100% divided by the total number of cells/field in 10 random fields at 400x magnification.

### 4.14. Statistics

All statistical analyses were carried out with SPSS 13.0 (SPSS Inc., Chicago, IL). The data values were presented as mean ± SD. All experiments were performed with triplicate data points and at least 3 times. Differences in mean values between two groups were analyzed by two-tailed Student's *t* test, and the mean values of more than two groups were compared with one-way ANOVA. *p* ≤ 0.05 was considered statistically significant.

## Data Availability

All data used to support the findings of this study are available from the corresponding author (Shuyuan Yeh, shuyuan_yeh@urmc.rochester.edu) upon request.

## Supplementary Materials

Supplementary 1 Supplementary Table 1: sequences of primers targeting genes for Q-PCR.

Supplementary 2 Supplementary Figure 1: depletion of renal ER*β* with 2nd shRNA made renal epithelial cells more vulnerable to oxalate-induced ROS production and cell injury. Figure 1S: A, the second shER*β* target sequence. B, qRT-PCR shows the ER*β* knockdown efficiency of the second shER*β* (shER*β*#2) in HK-2 cells. C, HK-2 cells were transduced with lentiviral shLuc (control) or shER*β*#2 and treated with 8% FBS-DMEM media or 8% FBS-DMEM media containing 0.75 mM oxalate for 6 hr; the ROS production in the cells was detected by dihydroethidium (DHE) staining under a fluorescence microscope. Representative DHE staining images are presented. Digital scans of DHE-stained cells were quantified using ImageJ software; *t*-test, compared to the shLuc group. D, detection of H_2_O_2_ levels in culture media of the shER*β*#2 or control (shLuc) renal tubular epithelial cells after challenge with 0.75 mM oxalate for 6 hr; Student's *t* tests, compared to the shLuc group. E, LDH release measurement in the ER*β* knocked-down HK-2 cells treated with 0.75 mM oxalate for 6 hr. For B-E, data are presented as mean ± SD. ^∗^ *P* < 0.05 and ^∗∗^ *P* < 0.01.

Supplementary 3 Supplementary Figure 2: generation and confirmation of the ER*β*KO mice. Figure 2S: generation and confirmation of the ER*β*KO mice. A, ER*β*KO mouse breeding scheme. B, tail genomic DNA was isolated for genotyping by PCR.
