# Supplementary material for: The Protective Roles of Estrogen Receptor β in Renal Calcium Oxalate Crystal Formation via Reducing the Liver Oxalate Biosynthesis and Renal Oxidative Stress-Mediated Cell Injury
Source: Oxid Med Cell Longev. 2019 Apr 17;2019:5305014. doi: 10.1155/2019/5305014 (PMC6501165; doi:10.1155/2019/5305014)
Supplement: Supplementary 1 — Supplementary Table 1: sequences of primers targeting genes for Q-PCR. [file 5305014.f1.docx]

Supplementary Table 1 - Sequences of primers targeting genes for Q-PCR

| **Species** | **Gene** | **Forward primer (5’ to 3’)** | **Reverse primer (5’ to 3’)** |
| --- | --- | --- | --- |
| Human | ERα | CCCACTCAACAGCGTGTCTC | CGTCGATTATCTGAATTTGGCCT |
| Human | ERβ | TCCATCGCCAGTTATCACATCT | CTGGACCAGTAACAGGGCTG |
| Human | GO | GGATGATGTGCGTAACAGATTCA | CAAGTCCACTGTCGTCTCCA |
| Human | AGT1 | GTCCATGAGCAAGGATATGTACC | GTCCCGAGCCAGAGATGAC |
| Human | AGT2 | TCCCGGACATCAGTAACCAAG | ACTGGTATCTTTCAGGCATGAAG |
| Human | p47phox | GGGGCGATCAATCCAGAGAAC | GTACTCGGTAAGTGTGCCCTG |
| Human | Rac1 | ATGTCCGTGCAAAGTGGTATC | CTCGGATCGCTTCGTCAAACA |
| Human | p67phox | CCAGAAGCATTAACCGAGACAA | CCTCGAAGCTGAATCAAGGC |
| Human | NOX2 | AACGAATTGTACGTGGGCAGA | GAGGGTTTCCAGCAAACTGAG |
| Human | p22phox | CCCAGTGGTACTTTGGTGCC | GCGGTCATGTACTTCTGTCCC |
| Human | NOX4 | TGTGCCGAACACTCTTGGC | ACATGCACGCCTGAGAAAATA |
| Human | OPN | CTCCATTGACTCGAACGACTC | CAGGTCTGCGAAACTTCTTAGAT |
| Human | MCP-1 | CAGCCAGATGCAATCAATGCC | TGGAATCCTGAACCCACTTCT |
| Human | Il-6 | ACTCACCTCTTCAGAACGAATTG | CCATCTTTGGAAGGTTCAGGTTG |
| Human | Gapdh | GGAGCGAGATCCCTCCAAAAT | GGCTGTTGTCATACTTCTCATGG |
