# Supplementary material for: The Protective Roles of Estrogen Receptor β in Renal Calcium Oxalate Crystal Formation via Reducing the Liver Oxalate Biosynthesis and Renal Oxidative Stress-Mediated Cell Injury
Source: Oxid Med Cell Longev. 2019 Apr 17;2019:5305014. doi: 10.1155/2019/5305014 (PMC6501165; doi:10.1155/2019/5305014)
Supplement: Supplementary 2 — Supplementary Figure 1: depletion of renal ERβ with 2nd shRNA made renal epithelial cells more vulnerable to oxalate-induced ROS production and cell injury. Figure 1S: A, the second shERβ target sequence. B, qRT-PCR shows the ERβ knockdown efficiency of the second shERβ (shERβ#2) in HK-2 cells. C, HK-2 cells were transduced with lentiviral shLuc (control) or shERβ#2 and treated with 8% FBS-DMEM media or 8% FBS-DMEM media containing 0.75 mM oxalate for 6 hr; the ROS production in the cells was detected by dihydroethidium (DHE) staining under a fluorescence microscope. Representative DHE staining images are presented. Digital scans of DHE-stained cells were quantified using ImageJ software; t-test, compared to the shLuc group. D, detection of H2O2 levels in culture media of the shERβ#2 or control (shLuc) renal tubular epithelial cells after challenge with 0.75 mM oxalate for 6 hr; Student's t tests, compared to the shLuc group. E, LDH release measurement in the ERβ knocked-down HK-2 cells treated with 0.75 mM oxalate for 6 hr. For B-E, data are presented as mean ± SD. ∗ P < 0.05 and ∗∗ P < 0.01. [file 5305014.f2.pptx]

## Slide 1
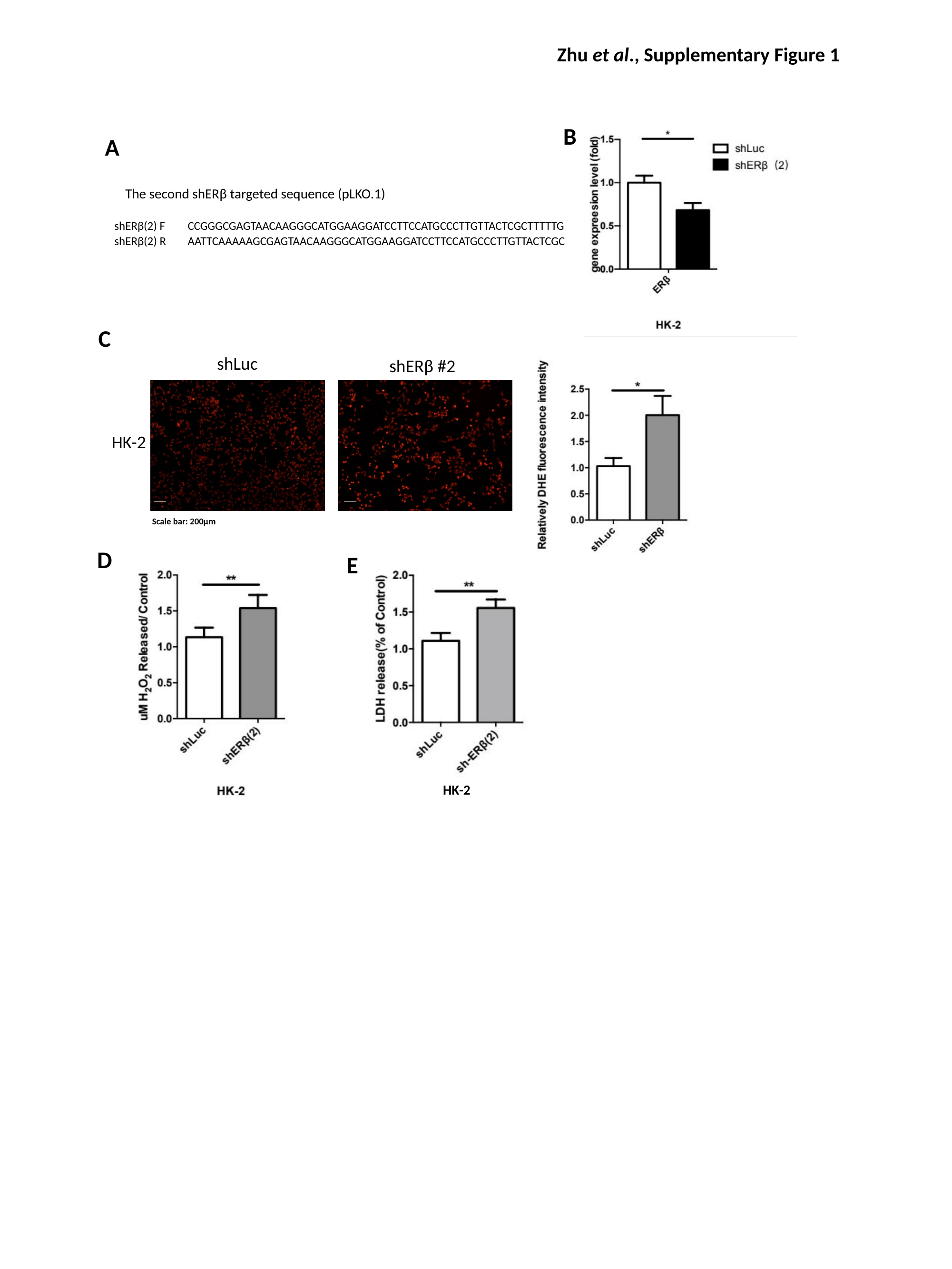

# Zhu et al., Supplementary Figure 1
B
A
The second shERβ targeted sequence (pLKO.1)
| shERβ(2) F | CCGGGCGAGTAACAAGGGCATGGAAGGATCCTTCCATGCCCTTGTTACTCGCTTTTTG |
| --- | --- |
| shERβ(2) R | AATTCAAAAAGCGAGTAACAAGGGCATGGAAGGATCCTTCCATGCCCTTGTTACTCGC |
C
shLuc
shERβ #2
HK-2
Scale bar: 200μm
D
E
HK-2
