# Supplementary material for: The Protective Roles of Estrogen Receptor β in Renal Calcium Oxalate Crystal Formation via Reducing the Liver Oxalate Biosynthesis and Renal Oxidative Stress-Mediated Cell Injury
Source: Oxid Med Cell Longev. 2019 Apr 17;2019:5305014. doi: 10.1155/2019/5305014 (PMC6501165; doi:10.1155/2019/5305014)
Supplement: Supplementary 3 — Supplementary Figure 2: generation and confirmation of the ERβKO mice. Figure 2S: generation and confirmation of the ERβKO mice. A, ERβKO mouse breeding scheme. B, tail genomic DNA was isolated for genotyping by PCR. [file 5305014.f3.pptx]

## Slide 1
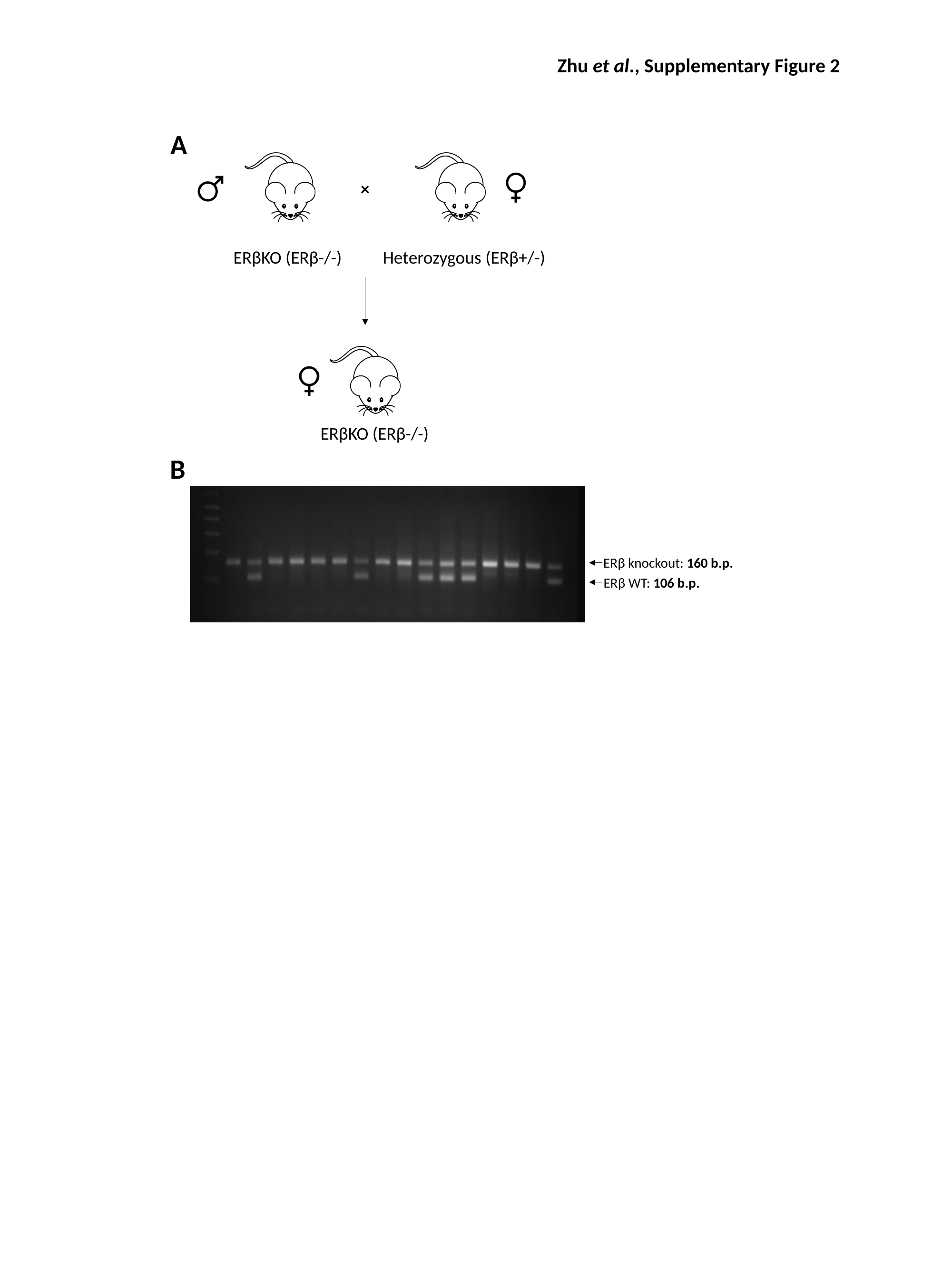

# Zhu et al., Supplementary Figure 2
A
×
ERβKO (ERβ-/-)
Heterozygous (ERβ+/-)
ERβKO (ERβ-/-)
B
ERβ knockout: 160 b.p.
ERβ WT: 106 b.p.
